# Integrating machine learning and bioinformatics analysis to m6A regulator-mediated methylation modification models for predicting glioblastoma patients’ prognosis and immunotherapy response

**DOI:** 10.18632/aging.204495

**Published:** 2023-05-23

**Authors:** Chuanyu Li, Wangrui Liu, Chengming Liu, Qisheng Luo, Kunxiang Luo, Cuicui Wei, Xueyu Li, Jiancheng Qin, Chuanhua Zheng, Chuanliu Lan, Shiyin Wei, Rong Tan, Jiaxing Chen, Yuanbiao Chen, Huadong Huang, Gaolian Zhang, Haineng Huang, Xiangyu Wang

**Affiliations:** 1 Department of Neurosurgery, Affiliated Hospital of Youjiang Medical University for Nationalities, Baise 533000, Guangxi, China; 2 Department of Interventional Oncology, Renji Hospital, Shanghai Jiao Tong University School of Medicine, Shanghai 200127, China; 3 Department of Neurosurgery, The First Affiliated Hospital of Soochow University, Suzhou 215006, Jiangsu, China; 4 Department of Outpatient, Affiliated Hospital of Youjiang Medical University for Nationalities, Baise 533000, Guangxi, China; 5 Department of Neurosurgery, The First Affiliated Hospital of Guangxi University of Chinese Medicine, Nanning 530023, Guangxi, China; 6 Department of Neurosurgery, The First Affiliated Hospital of Jinan University, Guangzhou 510632, Guangdong Province, China

**Keywords:** m6A, prognosis, consistent clustering, metastasis, glioblastoma

## Abstract

Background: Epigenetic regulations of immune responses are essential for cancer development and growth. As a critical step, comprehensive and rigorous explorations of m6A methylation are important to determine its prognostic significance, tumor microenvironment (TME) infiltration characteristics and underlying relationship with glioblastoma (GBM).

Methods: To evaluate m6A modification patterns in GBM, we conducted unsupervised clustering to determine the expression levels of GBM-related m6A regulatory factors and performed differential analysis to obtain m6A-related genes. Consistent clustering was used to generate m6A regulators cluster A and B. Machine learning algorithms were implemented for identifying TME features and predicting the response of GBM patients receiving immunotherapy.

Results: It is found that the m6A regulatory factor significantly regulates the mutation of GBM and TME. Based on Europe, America, and China data, we established m6Ascore through the m6A model. The model accurately predicted the results of 1206 GBM patients from the discovery cohort. Additionally, a high m6A score was associated with poor prognoses. Significant TME features were found among the different m6A score groups, which demonstrated positive correlations with biological functions (i.e., EMT2) and immune checkpoints.

Conclusions: m6A modification was important to characterize the tumorigenesis and TME infiltration in GBM. The m6Ascore provided GBM patients with valuable and accurate prognosis and prediction of clinical response to various treatment modalities, which could be useful to guide patient treatments.

## INTRODUCTION

Glioma is the highest incident primary brain tumor, accounting for ~80% of all adult brain cancer cases [1]. Glioblastoma multiforme (GBM) is classified as a grade IV cancer by the World Health Organization (WHO) and comprises 57.3% of all gliomas [2]. Its clinical presentation varies depending on its location and size and can be characterized by headaches, seizures, neurological dysfunctions, etc. [3]. Currently, little is known about its etiology [4]. Although multiple treatments have been developed for treating the patients, including surgery, chemotherapy, radiation and others, their survival rate remains far from satisfactory [5]. In addition, despite the promising efficacies of immunotherapy, most patients do not respond well [6, 7], urging the need for better treatments [8].

GBM develops in a highly complex tissue environment and depends on the continuous growth, invasion and metastasis of these environments, which is why it is constantly infiltrating and challenging to cure. Targeting the tumor microenvironment (TME) was shown to potentially decrease the risk of drug resistance and disease recurrence. TME has a variety of capabilities to induce the beneficial and unfavorable consequences of tumorigenesis, so how to destroy and promote the occurrence of GBM The TME is a challenging job [9–11].

N6-methyladenosine (M6A) methylation, a commonly observed modification on RNAs, is similar to DNA and histone modifications [12–15]. Its involvement has been reported in all the RNA stages life cycle, including regulation of RNA processes, translation, RNA degradation etc. [16–19]. Although recent discoveries indicated its association with cancer occurrence and development [17, 20, 21], its actual roles and underlying mechanisms remain unknown.

Recent literature reported that m6A was related to immunotherapy, which affects TME and its related immune cells by regulating targeted RNA. Therefore, m6A has become a potential target for immunotherapy. m6A may have different roles in different tumors, but the m6A detector has been shown to have broad significance in specific cancers [22–24]. In several cancers, including kidney, lung, gastric and other cancers [25–31], m6A-related signals have been identified as tumor immunity Phenotype and biomarker of anti-PD-1 immunotherapy response. These findings indicated that M6A modifications could mirror the TME status and anticipate immunotherapeutic effects in numerous cancers, not limited to specific cancer types. Another study found that m6A is inseparable from low-grade gliomas [32]. However, due to technical limitations, most studies focused on 1 or 2 m6A-related genes. TME is characterized by multiple highly coordinated components in a network, so there is no research on combining m6A and TME. In addition, deeper investigations on m6A methylation modification in GBM are needed to improve treatment outcomes.

In this study, we used 19 GBM-related m6A regulatory factors to analyze m6A modification patterns in GBM samples from the Chinese Glioma Genome Atlas (CGGA; http://www.cgga.org.cn/) and Cancer Genome Atlas (TCGA) databases, which were then validated in our own cohort. Next, we proposed a nomogram for quantifying the m6A modification patterns of GBM patients and predicting their potential immunotherapy response and prognosis. Altogether, our results showed that m6A modification promoted GBM progression and the potential clinical significance of our scoring system to guide the treatment and estimate the survival of GBM patients.

## MATERIALS AND METHODS

### Data

The mRNA expression profile data and sample copy number variation (CNV) of Caucasian GBM samples are downloaded from the University of California, Santa Cruz (https://xenabrowser.net/datapages/). The expression profiles of Chinese GBM samples were obtained at the CGGA. In addition, were downloaded from The GEO database (https://www.ncbi.nlm.nih.gov/geo/) were queried to obtain the expression profiles of three groups of glioblastoma samples, namely GSE83300 [33], GSE74187 [34, 35]. For clinical information and data, the R package cgdsr and [36] are used for processing. Table 1 shows the retrieved information from the datasets. Perform consistency processing on the above data, including z-score [37] and batch correction [38, 39].

**Table 1:** The retrieved information from the datasets.

**Data set**	**Classification**	**Number of samples**
TCGA	mRNA-seq	15
	AgilentG4502A_07_1	99
	AgilentG4502A_07_2	460
CGGA	CGGA.mRNAseq_325	139
	CGGA.mRNAseq_693	249
	CGGA.mRNA_array_301	84
GEO	GSE83300	50
	GSE74187	60
	GSE43378	50

### Unsupervised clustering

Expression data on 21 m6A regulators was extracted from 1,206 data in all data sets to determine m6A modification patterns. Some data set did not detect the expression of IGF2BP1 and METTL14, so the final expression extracted was 19 regulatory factors. These 19 m6A regulatory factors comprised seven writers (METTL3, RBM15, RBM15B, WTAP, KIAA1429, CBLL1 and ZC3H13), two erasers (ALKBH5 and FTO) and ten readers (YTHDC1, YTHDC2, YTHDF1), YTHDF2, NNPHHDF2MR, LRPPRC, ELAVL1), and based on their expression levels, various m6A modification patterns were determined and patients’ data were also analyzed [40].

### Gene set variation analysis (GSVA) and single sample gene set enrichment analysis (ssGSEA)

GSVA enrichment analyses were conducted for determining the difference of m6A modification mode using c2.cp.kegg.v6.2 from the MSigDB database (https://www.gsea-msigdb.org/gsea/index.jsp) [41]. ssGSEA analyses were conducted to determine the ratio of 24 immune cells in GBM [42], the Wilcox test for comparing different samples, and Cox regression analysis for comparing their survival.

### Differentially expressed genes (DEGs) between m6A clusters

The TCGA, CGGA and GEO datasets were grouped into two categories according to the 19 m6A gene expressions (Supplementary Tables 1, 2). Using the R “limma” package, we identified DEGs among the different m6A clusters [43] based on *P*<0.05 and difference multiple >1.5 times or <0.67 times.

### m6A score calculation

Using the DEGs identified above, we implemented the random forest method to eliminate redundancy and perform survival analysis (P value <0.05). The DEGs were classified into two groups (positive or negative coefficients) through cox regressions. Refer to GGI scoring based on the equation to calculate m6Ascore [44].


m6Ascore=scale (∑X−∑Y)

Scale represents the standardization process, while X and Y represent the gene set expressions using a positive and negative Cox coefficient, respectively.

surv_cutpoint function was used for identifying the best threshold point (cutoff=-0.9884624) [45] to divide the samples into a high and low m6A score, performing correlation analysis and survival estimation.

### Correlation between m6A score and other biological processes

GSVA analyses were conducted for quantifying the biological functions of the samples, and Pearson correlation analysis on the m6Ascore and ES scores of these biological functions to reveal underlying biological pathways involved in m6Ascore.

GISTIC (Q≤0.05, 95%CI) was computed to determine the common copy number change area in the samples [46].

The R “pRRophetic” package was used to obtain the IC50 of Cisplatin and Gemcitabine based on the expression profiles and to compare the difference in IC50 between the high and low m6A scores.

The TIDE tool (http://tide.dfci.harvard.edu/) was used to assess the clinical effect of immunotherapy. A higher TIDE score indicated poor immunotherapy efficacy and prognosis. Among five cancers with immune dysfunctions and rejection characteristics, only melanoma cases treated with anti-PD1 or anti-CTLA4 were available in public databases. Immune checkpoint treatment prognosis prediction in this analysis is completed by TIDE score.

### Consent for publication

All authors agree to publish.

## RESULTS

### Genetic variation of m6A regulatory factor of GBM in TCGA database

**Figure 1:**
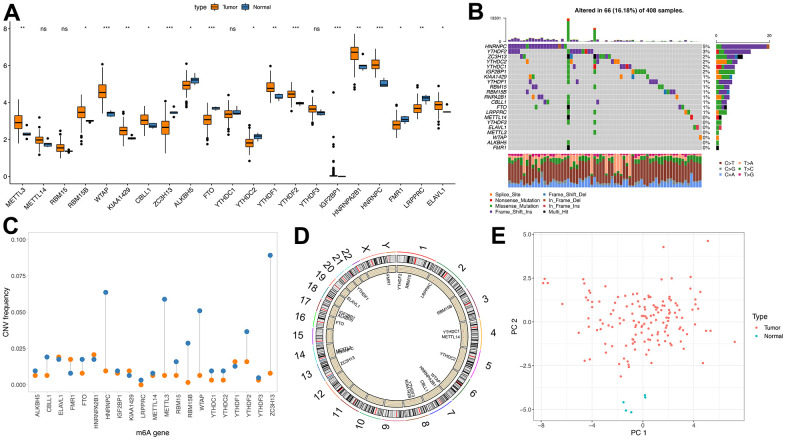
**Genetic variation of m6A regulator.** (**A**) m6A expression of tumor and normal samples in TCGA; (**B**) Distribution of m6A gene mutations and different mutation types; (**C**) CNV incidence of m6A gene, blue indicates deletion and orange indicates amplification; (**D**) Position of m6A gene on chromosome; (**E**) PCA results of m6A gene in TCGA samples).

Here, we aimed to investigate the m6A regulators’ genetic backgrounds and variations in GBM. First, we selected all tumor samples in the TCGA database and analyzed the 21 m6A regulators, the eight writers, two erasers and 11 readers. First, we studied the mRNA levels of the m6A regulatory factors between TCGA-derived tumors and normal samples, which showed greater expressions in the tumor samples, including METL3 and WTAP (p<0.01) (Figure 1A). This shows that the role of m6A, in general, is to promote tumor growth.

Then, the CNV and somatic mutation rate of 20 m6A regulatory factors in GBM samples were investigated. HNRNPC has the highest mutation frequency, reaching 5% (Figure 1B). CNV mutations are generally changed in regulatory factors. The copy number of some genes is amplified. The frequency of CNV deletion is noticeable in genes such as HNRNPC, METL3 and ZC3H13 (Figure 1C and Supplementary Tables 3, 4). In addition, m6A regulated The position of the organ on chromosomes (Figure 1D). PCA analysis showed that tumors and normal samples could be distinguished (Figure 1E).

### Unsupervised clustering of m6A genes in GBM samples

**Figure 2:**
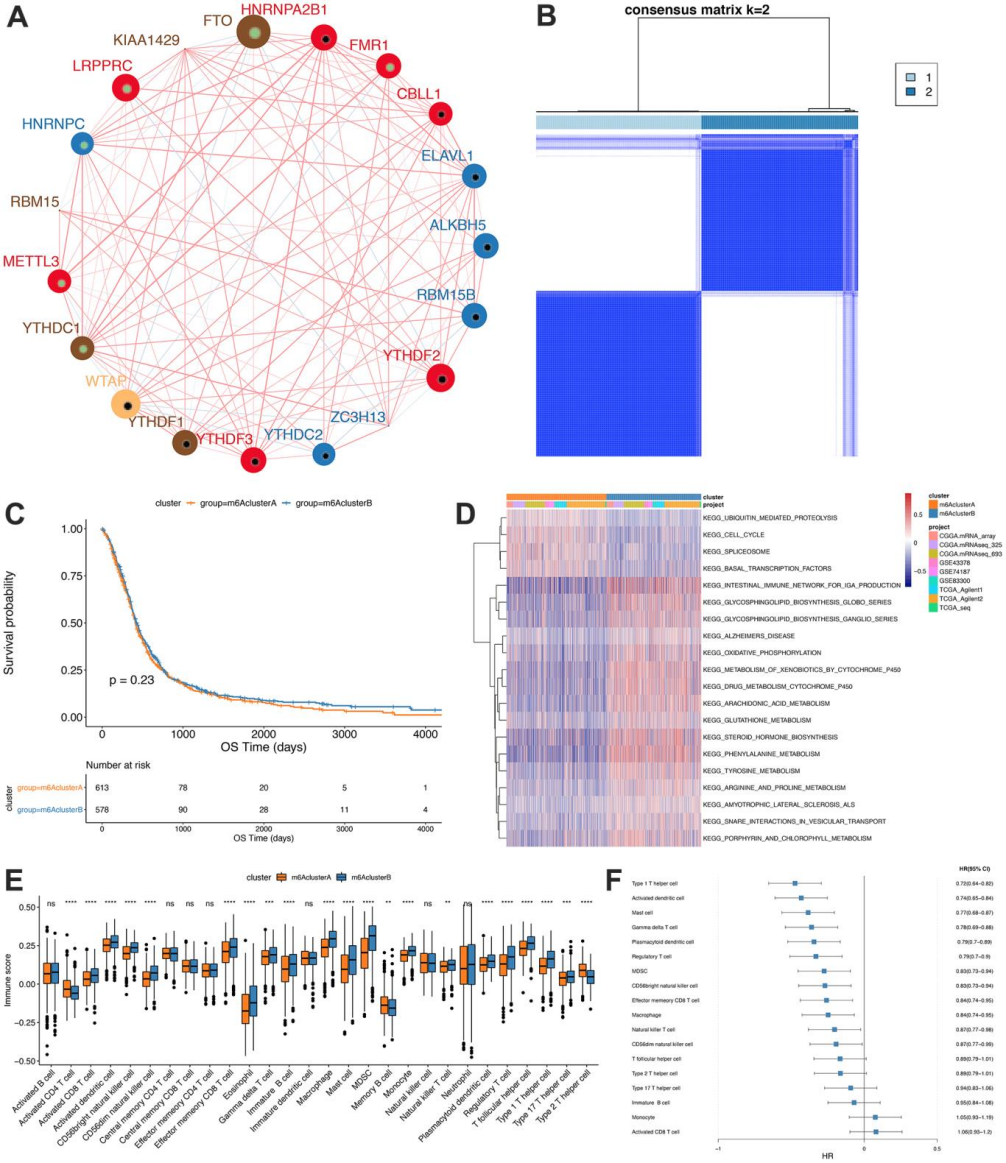
**Unsupervised clustering of m6A genes in low-grade glioma samples.** (**A**) Interaction between m6A genes. The size of the circle indicates the impact of each gene on survival prediction, and the larger the expression, the more relevant the prognosis. In the circle the green dots in the circle indicate prognostic protective factors, and the black dots in the circle indicate prognostic risk factors. The lines connecting genes show their interactions. The negative correlations are marked in blue and positive correlations in red. Gene clusters ABC are marked respectively in blue, red and brown; (**B**) Consistent clustering of m6A genes; (**C**) Kaplan-Meier curve showed no significant survival differences in two m6Aclusters; (**D**) GSVA enrichment analysis, showing the biological pathways with different m6Aclusters Activation state. Heat map is used to visualize these biological processes, red means activation and blue means inhibition; (**E**) Distribution of immune infiltration of 28 immune cells in two m6Aclusters; (**F**) Differential cell prognosis analysis.

Due to the lack of IGF2BP1 and METTL14 expression levels in some data sets, we determined the consistency clustering of the m6A genes and m6A gene single-factor Cox regression using gene expression profile data of 19 m6A regulators and the survival data from the TCGA, CGGA and GEO data sets. Findings from the m6A regulatory networks indicated the associations between the m6A regulatory factors (Figure 2A) and that between regulatory factors and prognosis. The influence of m6A regulators on patients’ survival was demonstrated in Supplementary Table 5_celluar-types.tsv. Moreover, we found that m6A of both the same functional category and different functional categories showed a significant correlation (spearman); the statistical results are shown in Supplementary Table 5_celluar -interactions. tsv.

Our findings indicated that interactions between m6A regulators of different functional categories played essential roles for forming different m6A modifications in GBM. Then, the expressions of the regulators from TCGA, CGGA and GEO were obtained, and we utilized the R “ConsensusClusterPlus” package to conduct consistency clustering, based on which m6A clusters A and B were identified (Figure 2B) and further analyses showed that the prognosis between them was similar (*P*=0.23, Figure 2C).

### Functional annotations and TME infiltration characterization of the m6A clusters

GSVA enrichment analysis was conducted to assess the differences between the biological behaviors of the regulatory factors in the two m6A modification subgroups. m6A cluster A was found to be significantly enriched in cell cycle pathways, while m6A cluster B in biosynthesis and oxidative phosphorylation of glycosphingolipids pathways (Figure 2D, Significant analysis results are shown in Supplementary Tables 6, 7).

Furthermore, ssGSEA analysis using the RNA-seq data was conducted to determine the proportions of 28 immune cells, such as B memory cells, activated Dendritic cells and M0 macrophages, in GBM samples (Figure 2E) and the findings showed significantly different distributions of immune cells abundance between the two subgroups. Figure 2F shows the results of single-factor Cox regression analysis of different proportions of immune cells between m6A clusters A and B (Analysis results are shown in Supplementary Table 8).

**Figure 3:**
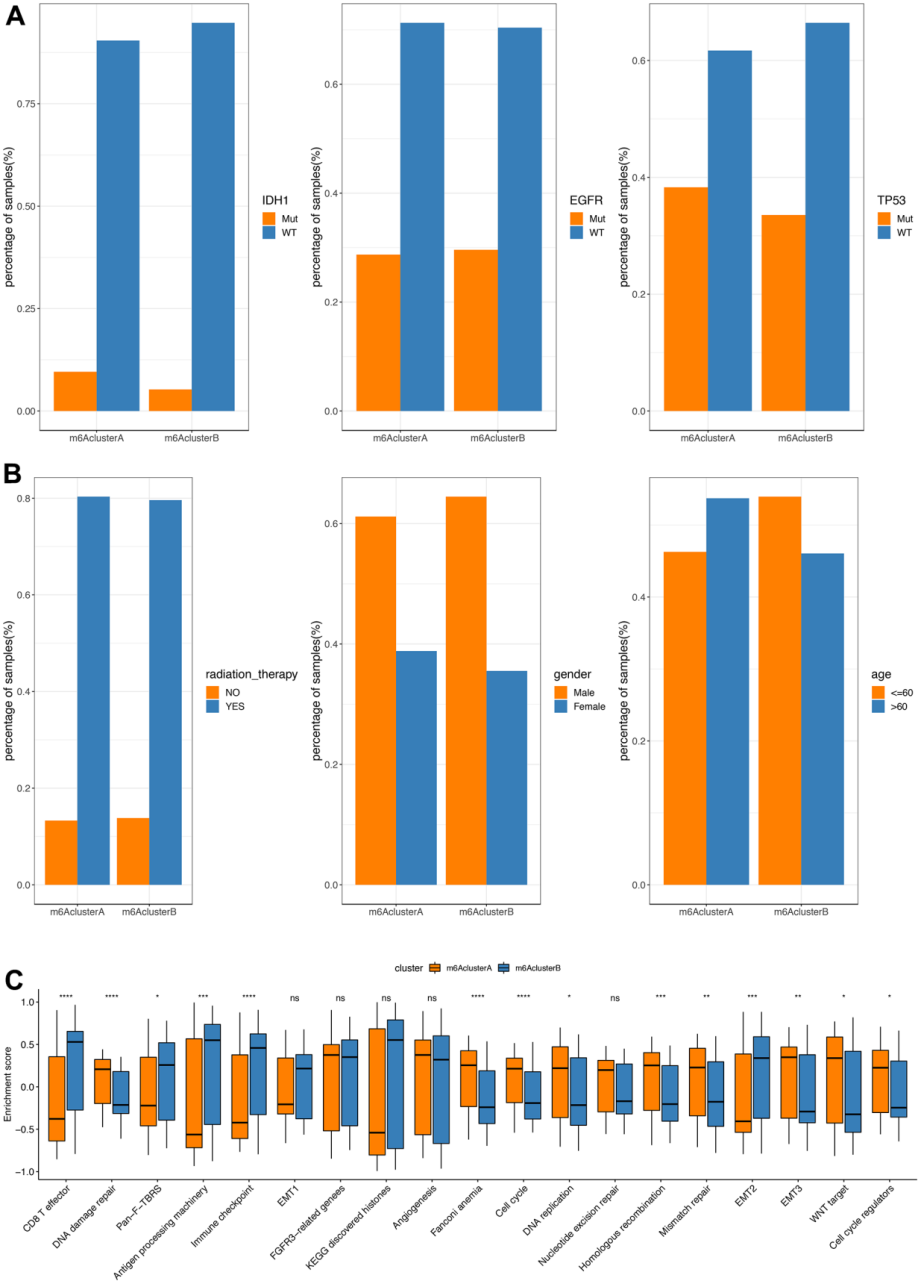
**Comparative analysis between m6Acluster in the TCGA dataset.** (**A**) The distribution of IDH1, EGFR, TP53 mutations in the 2 m6Aclusters; (**B**) The distribution of radiotherapy, gender, and age in m6Acluster; (**C**) The enrichment scores of different m6Acluster groups difference (**P<0.05, *** *P*<0.01, ****P<0.001).

In the TCGA database, we found that the mutation difference of IDHI (chi-square test, *P*=0.1998181), TP53 (chi-square test, *P*=0.4284502) and EGFR (chi-square test, *P*=0.9538232) in subgroups m6AclusterA and m6AclusterB were not significant (Figure 3A, specific information is shown in Supplementary Table 9). No obvious differences were observed between their clinical characteristics (Figure 3B).

**Figure 4:**
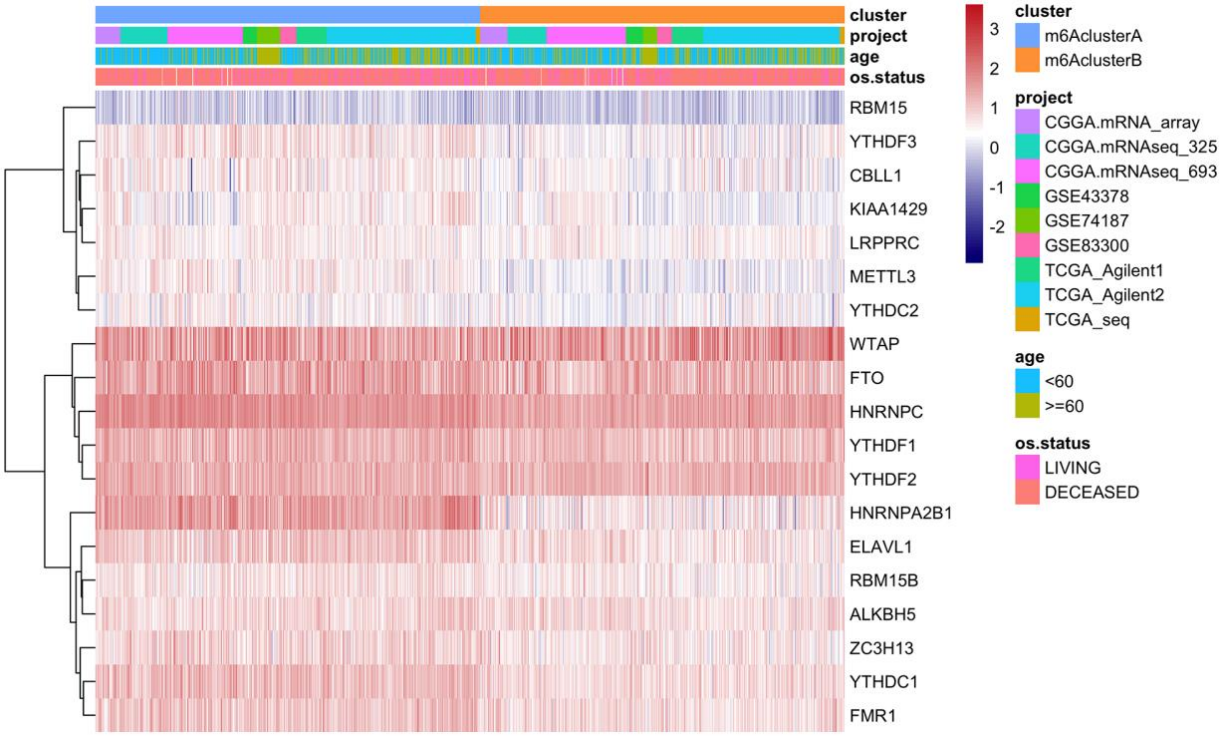
The expression of m6A regulatory factors in m6Acluster.

GSVA analysis was carried out with gene set constructed by Mariathasan; there is a significant difference of enrichment scores between the m6Acluster groups (Figure 3C, analysis results are shown in Supplementary Table 10). Additionally, the m6A regulatory factor distributions in m6AclusterA and m6AclusterB are shown in Figure 4 (Analysis results are shown in Supplementary Table 11).

### m6A-related DEGs and constructions of the m6Agenecluster

The biological behaviors of each m6Acluster were detected in 73 phenotype-related differential genes with a limma software package (Supplementary Table 12).

**Figure 5:**
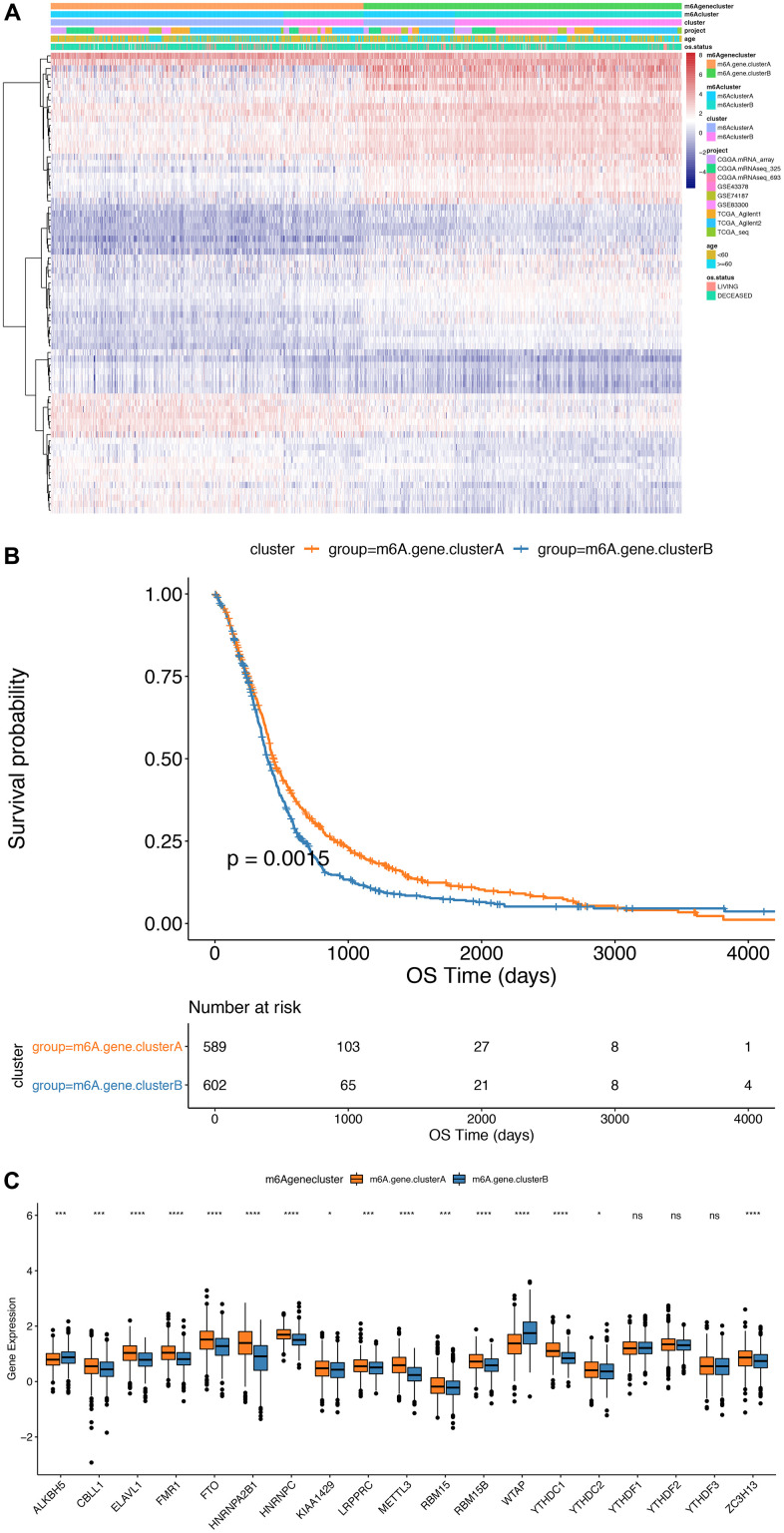
**Comparison between m6Agenecluster.** (**A**) Unsupervised clustering of m6A phenotype-related genes in low-grade glioma samples. The samples are divided into different genomic subtypes, called m6AgeneclusterA and m6AgeneclusterB; (**B**) Kaplan-Meier curve indicates that m6A modifies the genome table Type has an obvious relationship with overall survival rate; (**C**) Expression of 19 m6A genes in 2 gene clusters. The upper and lower ends of the box indicate the interquartile range of values. The line in the box indicates the median value, and the black dots indicate outliers. The T test is used to test the statistical differences between gene clusters (**P<0.05, ***P<0.01, ****P<0.001).

Furthermore, we implemented an unsupervised cluster analysis using the phenotype-related genes of m6A to classify patients into separate genomic subgroups according to different genomic subtypes based on their m6A-modified genomic phenotypes, m6A gene cluster A and m6A gene cluster B (Figure 5A and Supplementary Table 13). The findings showed the prognosis of m6A gene cluster A tumor was better than m6A gene cluster B, and part of the m6A regulatory factors expression levels were markedly greater compared to m6A gene cluster B (Figure 5C).

### Analysis of m6Ascore

**Figure 6:**
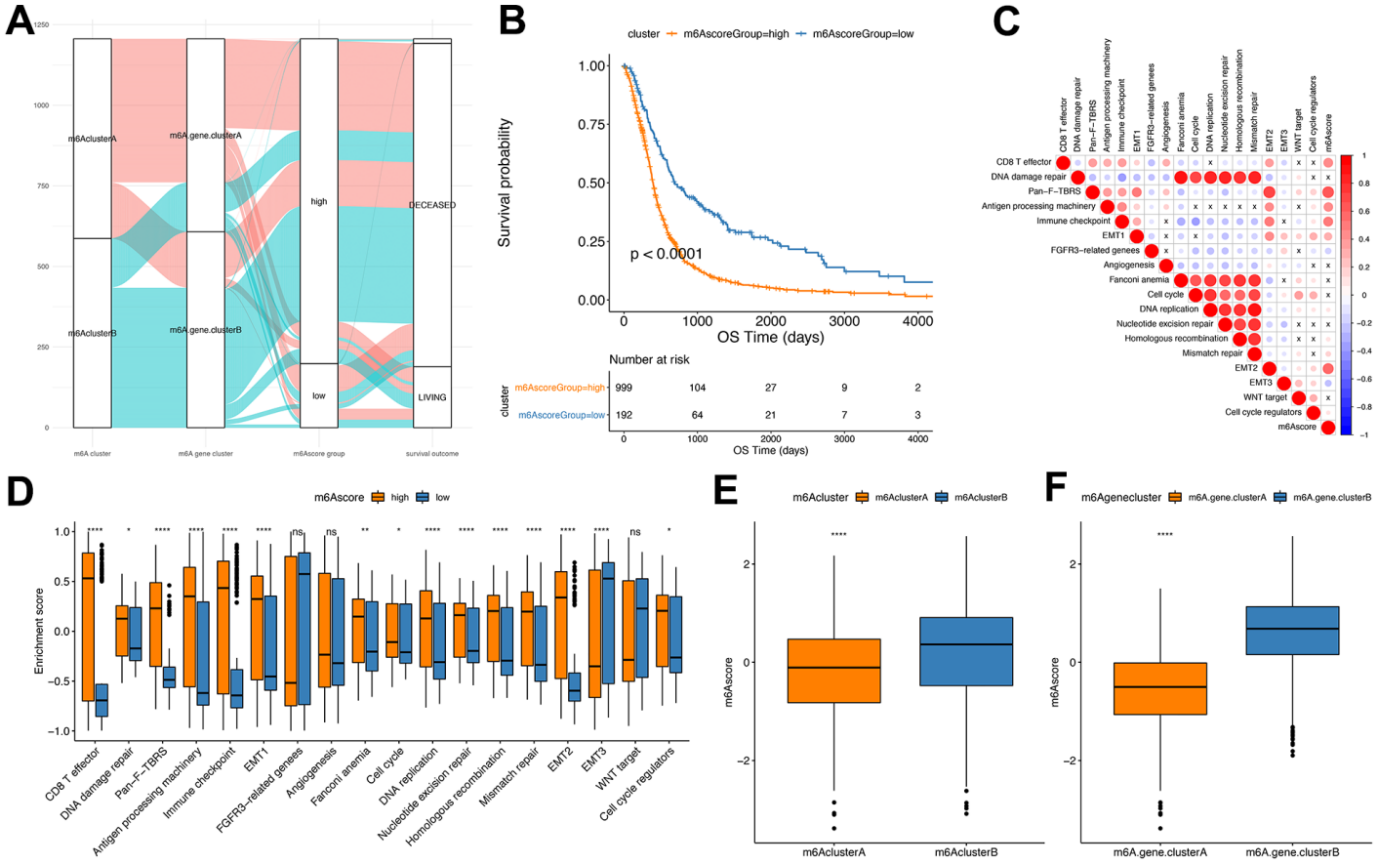
**Establishment of m6Ascore.** (**A**) Alluvial plot showing the changes of m6A cluster, gene cluster and m6Ascore; (**B**) Kaplan-Meier curve shows that m6Ascore high and low grouping has a significant relationship with overall survival rate; (**C**) Using Pearson analysis, the correlation between m6Ascore and known gene features in GBM. Negative correlation is marked in blue, and it is positively correlated with red. X in the figure indicates that the correlation is not significant, and the larger the circle, the more significant; (**D**) The distribution of the enrichment scores of known gene features in the m6Ascore high and low group samples in the TCGA+CGGA+GEOdata set (***P<0.01, ****P<0.001); (**E**) The distribution of m6Ascore in m6Acluster (****P<0.001); (**F**) Distribution of m6Ascore in m6Agenecluster (****P<0.001).

The Random Forest method was implemented to remove the redundancy in the DEGs and identified significant genes associated with the classification (Supplementary Table 14). Cox regression was performed to establish the association between the significant genes and GBM patients’ survival. The genes’ coefficient values were used to separate them into two groups, and all samples were scored with the m6Ascore formula. Finally, based on the surv_cutpoint function of R package survminer, the optimal threshold of m6score was determined (cutoff=-0.9884624) as a standard to divide the samples into m6Ascore^high^ group and m6Ascore^low^ group (Figure 6A and Supplementary Table 16). Our results indicated that the m6Ascore^low^ group had significantly better survival than the m6Ascore^high^ group (*P*<0.0001), suggesting that the m6Ascore could be used to accurately characterize GBM patients’ prognoses (Figure 6B).

Correlation assessment between m6Ascores and known gene features indicated a significant positive correlation between m6Ascore and biological functions like EMT2 and immune checkpoint (Figure 6C and Supplementary Table 15). Wilcox test showed significant differences between m6Acluster and m6Agenecluster in m6ascore (Figure 6E, 6F). Further, the m6AclusterA and m6AgeneclusterA had significantly superior m6Ascore compared with the other groups.

Furthermore, in the TCGA cohort, a significant difference in m6A scores between the subgroups, such as *IDH1* mutation status and T*P53* mutations, were found.

**Figure 7:**
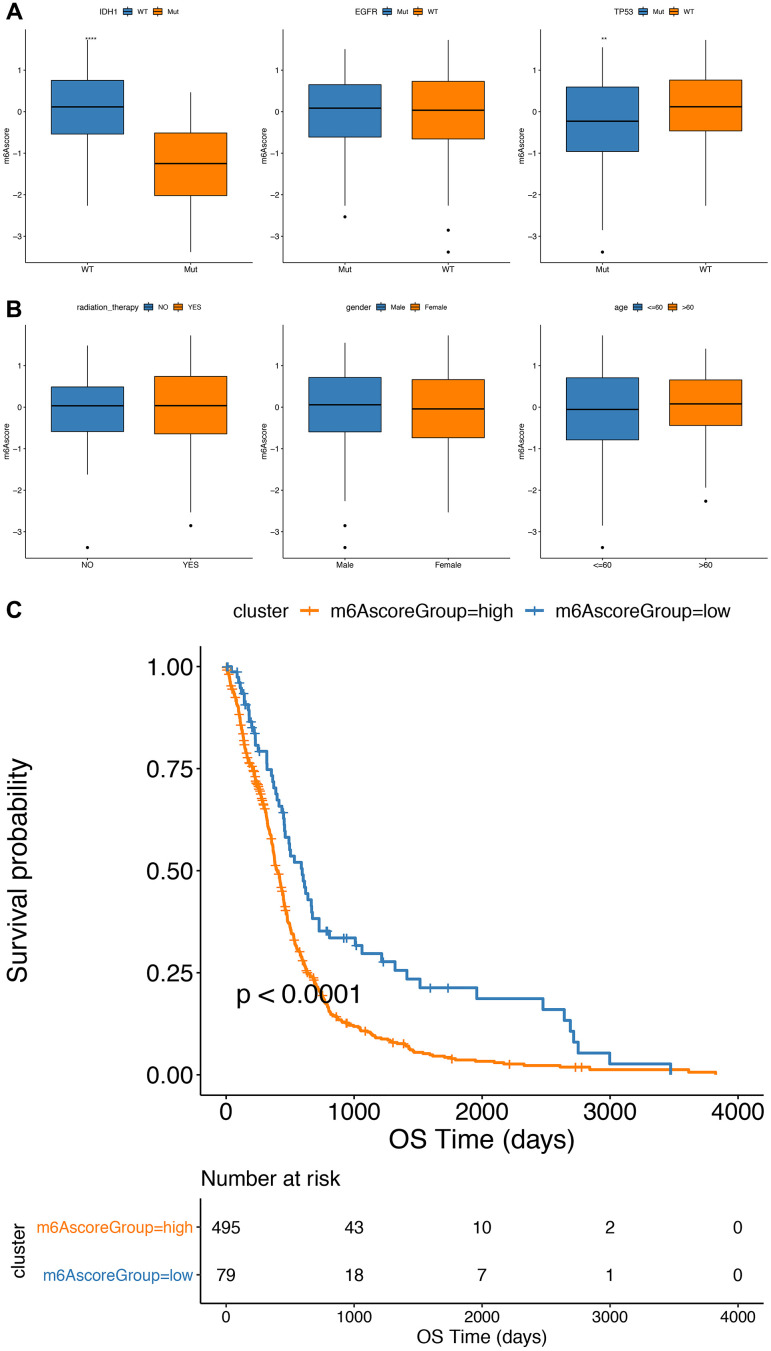
**Comparative analysis and model verification of m6Ascore in TCGA dataset.** (**A**, **B**) The distribution of m6Ascore in different subgroups; (**C**) there was significant difference in survival between m6Ascore^high^ group and m6Ascore^low^ group in TCGA samples.

Statuses, Wilcox test *P* value was 1.4e-09 and 0.0015) (Figure 7A, 7B). In addition, the m6Ascore^low^ subgroup had a significantly better prognosis than the m6Ascore^high^ subgroup of GBM (*P*<0.0001; Figure 7C).

### Differential molecular characteristics in m6Ascorehigh and m6Ascorelow group

**Figure 8:**
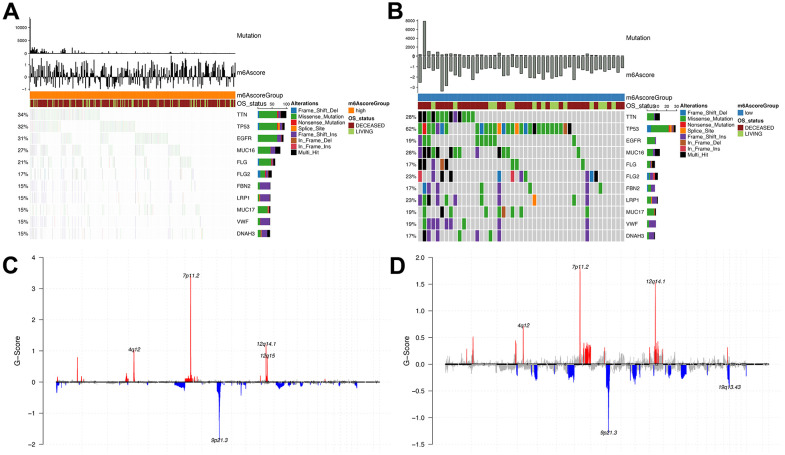
**Analysis of molecular characteristics of m6Ascore high and low groups.** (**A**, **B**) Distribution of gene mutations in samples of m6Ascore high and low groups; (**C**, **D**) The distribution of copy number amplification and deletion regions in the sample set of m6Ascore high and low groups.

Here, we compared the m6Ascore^high^ with the m6Ascore^low^ groups in the TCGA dataset. Differences in somatic mutations were determined using the R “maftools” package. Significant alterations were observed in the frequency of TTN (m6Ascorehigh, 34%; m6Ascorelow, 28%), TP53 (m6Ascorehigh, 32%; m6Ascorelow, 62%) and MUC16 (m6Ascorehigh, 27%; m6Ascorelow, 28%) genes (Figure 8A, 8B). Figure 8C, 8D shows the distribution in CNV regions between the m6Ascorehigh and m6Ascorelow groups.

### m6Ascore predicted chemotherapy and immunotherapy responses of GBM patients

**Figure 9:**
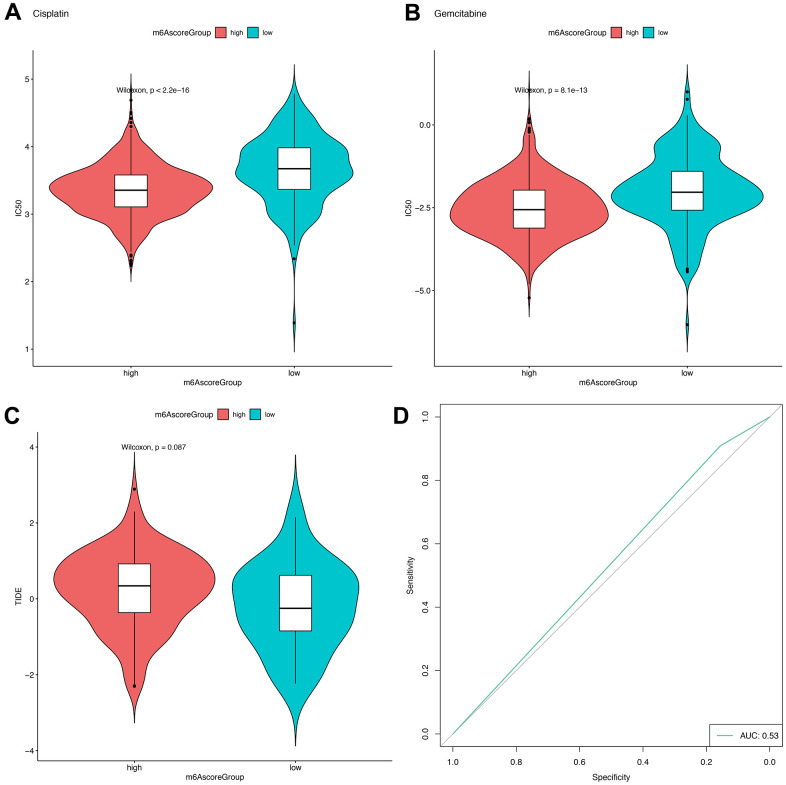
**m6Ascore provided predictive outcomes for GBM patients receiving immunotherapies.** (**A**, **B**) The difference between the IC50 values of Cisplatin and Gemcitabine in the samples of the high-risk group and the low-risk group; (**C**) The difference of TIDE score between samples of high-risk group and low-risk group; (**D**) Prediction of immunotherapy response results by using high and low groups of m6Ascore.

In TCGA, CGGA and GEO datasets, we further estimated and compared the IC50 values of Cisplatin and Gemcitabine according to the expression levels of the m6Ascorehigh and m6Ascorelow groups using the R “pRRophetic” package, and found that a significantly greater IC50 value in the m6Ascorelow group compared with the m6Ascorehigh group, which suggest that patients in the m6Ascore^high^ group had poorer drug resistance (Cisplatin: *P*<2.2e-16, Gemcitabine: *P*=8.1e-13; Figure 9A, 9B).

Meanwhile, in 154 samples with sequencing data (including 139 samples with chip data) from the TCGA database, a numerically greater TIDE score was observed for the m6Ascorehigh group compared with the m6Ascorelow group (Figure 9C), but the difference is not significant. The AUC of the ROC curve for the m6score in predicting immunotherapy response was 0.53.

## DISCUSSION

Multimodal therapies are the standard treatments for GBM, which include surgery resection, radiation therapy, and systemic treatment with temozolomide [47]. However, traditional treatment methods often have serious side effects, and the treatment effect is not very good, thereby cannot significantly improve patients’ survival [48]. We explored the molecular and clinical characteristics between different subgroups based on the 19 m6A genes through consistent clustering. Further, by dimensionality reduction in m6A-related DEGs between the subgroups, we determined their association with patient prognosis. Then m6Ascore of these genes were calculated, and the differences between the m6Ascore^high^ group and m6Ascore^low^ group were compared.m6Ascore based classification was used for prediction of immunotherapy response. There exist some advantages of this study. Significant differences are shown in prognosis, clinical features or molecular characteristics between the groups of m6Agenecluster and m6Ascore. There are also some limitations. First, in the prediction of immunotherapy response, AUC of ROC curve is comparably low, only reached 0.53. Second, the difference of m6Acluster in prognosis and clinical characteristics were not significant. The clinical features of this part of glioblastoma did not find the status information of 1p19q. By consulting the data, this feature belongs to GBM.

In the past decades, many new concepts were investigated as an attempt to improve the treatments of GBM, including genetic testing, electromagnetic field therapy, and function-guided resection [2, 49]. However, the underlying causes of GBM malignant progression have not been explored, so there are still no biomarkers to accurately predict the prognosis and exacerbation of GBM patients [32, 50].

The TME and purity of tumor cells have important roles in tumor invasion and progression [51]. Therefore, by comprehensively analyzing the properties of TME and the cells it recruits in GBM, the immunophenotype of GBM at various stages can be identified, so as to find accurate biomarkers and discover new and effective therapeutic targets [52, 53].

Determining the roles and underlying mechanisms of m6A modification is a hot topic in cancer research and recent investigations showed that m6A regulators can regulate various tumor progression processes [11, 54]. Based on the limitations of previous related studies our group’s previous studies reported that the key markers and TME of LGG and GBM are not the same. In previous studies, the research group has found that m6Ascore accurately predicted LGG progression. Thus, in this present study, we carried out a precise analysis of GBM and found that the m6Acore could predict GBM patients’ prognosis and immunotherapy effects. These findings could be used as references to further determine the GBM’s etiology and assess more beneficial treatments.

## CONCLUSIONS

Our findings showed that m6A modification played pivotal roles in the tumorigenesis and TME infiltration characterization of GBM using data from large cohorts. Our proposed m6Ascore accurately predicted patients’ prognosis and potential chemotherapy and immunotherapy responses, providing novel perspectives to deepen our understanding on the pathogenesis of GBM and identify potential targets to improve treatment outcomes.

## Supplementary Material

Supplementary Table 1

Supplementary Table 2

Supplementary Table 3

Supplementary Table 4

Supplementary Table 5a

Supplementary Table 5b

Supplementary Table 6

Supplementary Table 7a

Supplementary Table 7b

Supplementary Table 8

Supplementary Table 9

Supplementary Table 10

Supplementary Table 11a

Supplementary Table 11b

Supplementary Table 12

Supplementary Table 13

Supplementary Table 14

Supplementary Table 15

Supplementary Table 16
