# Supplementary material for: Integrating machine learning and bioinformatics analysis to m6A regulator-mediated methylation modification models for predicting glioblastoma patients’ prognosis and immunotherapy response
Source: Aging (Albany NY). 2023 May 23;15(10):4051–70. doi: 10.18632/aging.204495 (PMC10257999; doi:10.18632/aging.204495)
Supplement: Supplementary Table 12 [file aging-15-204495-s015.pdf]

Supplementary Table 12. DEGm6Acluster.

HNRNPA2B1
ZNF146
GPSM3
RILP
EBI3
SPNS3
TCF12
SIGIRR
FUS
LY86
NOTCH1
GCHFR
SMC4
GMFG
ZNF711
PYCARD
REV3L
SELM
DUSP23
CHD7
SOX4
TRIB2
ETV1
VAMP5
VAMP8
CD86
RARRES3
LST1
SERPINB1
GLCCI1
IGFBP6
MAGI1
CFD
CENPF
SPAG4
VMO1
AIF1
CD33
COPZ2
IL21R
MEX3A
PPAP2C
KIF15
PPP1R14A

RNASE1
NUSAP1
GAS1
HSPB2
PTPRZ1
MT1E
TOP2A
SOX11
MKI67
LRRN1
S100A9
LMNB1
EVI2A
MEX3B
MS4A4A
CH25H
PTGDS
RCAN2
AMICA1
LYVE1
CYB5R2
FBXO2
BIRC3
ANGPTL4
CCL2
CYP1B1
PDGFRA
CHI3L2
LTF
