# Supplementary material for: Integrating machine learning and bioinformatics analysis to m6A regulator-mediated methylation modification models for predicting glioblastoma patients’ prognosis and immunotherapy response
Source: Aging (Albany NY). 2023 May 23;15(10):4051–70. doi: 10.18632/aging.204495 (PMC10257999; doi:10.18632/aging.204495)
Supplement: Supplementary Table 14 [file aging-15-204495-s017.pdf]

Supplementary Table 14. Sig genes.

ZNF146
TCF12
SMC4
TRIB2
SERPINB1
GLCCI1
CENPF
SPAG4
KIF15
NUSAP1
MKI67
LRRN1
S100A9
AMICA1
BIRC3
ANGPTL4
CCL2
CYP1B1
CHI3L2
LTF
